# Perineuronal Nets and CNS Plasticity and Repair

**DOI:** 10.1155/2016/4327082

**Published:** 2016-02-16

**Authors:** Daniela Carulli, Jessica C. F. Kwok, Tommaso Pizzorusso

**Affiliations:** ^1^Department of Neuroscience, Neuroscience Institute of Turin (NIT), University of Turin, 10100 Turin, Italy; ^2^Neuroscience Institute of the Cavalieri-Ottolenghi Foundation (NICO), Regione Gonzole 10, Orbassano, 10043 Turin, Italy; ^3^Netherlands Institute for Neuroscience (NIN), 1105 BA Amsterdam, NetherlandsNetherlands; ^4^John van Geest Centre for Brain Repair, Department of Clinical Neuroscience, University of Cambridge, Forvie Site, Cambridge CB2 0PY, UK; ^5^School of Biomedical Sciences, Faculty of Biological Sciences, University of Leeds, Leeds LS2 9JT, UK; ^6^Department of Neuroscience, Psychology, Drug Research, and Child Health (NEUROFARBA), University of Florence, 50134 Florence, Italy; ^7^Institute of Neuroscience, CNR, Via Moruzzi 1, 56124 Pisa, Italy

The extracellular matrix (ECM) of the nervous system regulates numerous events during development, from neurogenesis and gliogenesis to circuitry formation, as well as in adulthood, affecting damage responses, plasticity, and regeneration. Substantial changes in the quantity and the composition of ECM occur during development. At the end of critical periods, that is, temporal windows during development when experience-dependent neuronal plasticity is heightened, a specialised ECM structure called perineuronal net (PNN) deposits around many types of neuron, helping in stabilizing the newly established neuronal connections. In recent years, several other functions of the PNNs have been revealed, including restriction of neuronal plasticity, neuronal protection, and modulation of the pathogenesis of various CNS diseases. Elucidating the mechanisms through which PNNs act is challenging but holds a tremendous therapeutic potential for treating several CNS conditions.

In this special issue, we collected research and review articles that focus on different aspects of PNN structure, development, and function in health and disease.

In the article “Development and Structural Variety of the Chondroitin Sulfate Proteoglycans-Contained Extracellular Matrix in the Mouse Brain,” N. Horii-Hayashi et al. provide the first systematic study of PNN formation at the level of the whole brain of the mouse, from postnatal (P) day 3 to 11 weeks. The spatiotemporal distribution of* Wisteria floribunda* agglutinin-binding PNNs is described in several brain regions, including the brainstem, hypothalamus, limbic regions, and cerebral cortex. The period of PNN formation differs among distinct brain areas, supporting the idea that PNN maturation is functionally related to the closure of critical periods for the acquisition of specific functions.

The study by A. L. Mueller and colleagues, entitled “Distribution of N-Acetylgalactosamine-Positive Perineuronal Nets in the Macaque Brain: Anatomy and Implications,” addresses the distribution of PNNs and the proportion of neurons surrounded by PNNs in different areas of the rhesus macaque CNS. Highly variable proportions of PNNs characterize the monkey CNS, being most abundant in the cerebellar nuclei and less abundant in the cerebral cortex and midbrain. PNNs were found around parvalbumin-positive as well as parvalbumin-negative neurons. A useful discussion is provided about PNN expression in the primate CNS compared to rodent and human brain, which suggests that PNN prevalence is broadly maintained across taxa.

In the review “Neuron-Glia Interactions in Neural Plasticity: Contributions of Neural Extracellular Matrix and Perineuronal Nets,” A. Faissner et al. show recent data on the role of PNNs in the context of astrocyte-neuron interactions and their regulatory function in the establishment, maintenance, and plasticity of synaptic connections. The impact of specific ECM components on the expression of PNNs, neuronal activity, synaptogenesis, and synapse stabilization is discussed. A comprehensive overview of PNN structure, cellular origin of PNN components, PNN binding partners, and main functions of PNNs in the regulation of plasticity (at the circuit, cellular, and synapse level) is also provided, together with a description of neurological conditions in which PNNs are altered.

The article “Reorganization of Synaptic Connections and Perineuronal Nets in the Deep Cerebellar Nuclei of Purkinje Cell Degeneration Mutant Mice” by M. Blosa et al. addresses the role of PNNs in the regulation of structural plasticity in the adult brain in a deafferentation model. By employing* pcd* mice, which display slow Purkinje cell degeneration during the late postnatal age, the authors show increased sprouting of glutamatergic afferents, paralleled by decreased expression of specific PNN components, in the denervated cerebellar nuclei. Based on their findings, an interesting discussion on the role of neuron-versus astrocyte-released PNN molecules is provided.

The condensation of chondroitin sulfate proteoglycans (CSPGs) into PNNs and, as a consequence, the termination of the critical period for ocular dominance plasticity in the mouse visual cortex depends on a developmental increase in the 4-sulfation/6-sulfation ratio of chondroitin sulfates in the CSPGs (Miyata et al., 2012, Nature Neuroscience). In the article “Chondroitin 6-Sulfation Regulates Perineuronal Net Formation by Controlling the Stability of Aggrecan,” S. Miyata and H. Kitagawa further extend our knowledge on this topic, showing that increased 6-sulfation leads to a decreased expression of the CSPG aggrecan, by accelerating ADAMTS-5-mediated aggrecan proteolysis.

Another key evidence demonstrating the significance of CS sulfation in regulating PNN functions is detailed in the review “Otx2-PNN Interaction to Regulate Cortical Plasticity” by C. Bernard and A. Prochiantz. The group has previously demonstrated that sulfation pattern is crucial for the interaction between CSPGs and one of its binding molecules, the homeoprotein Otx2. Otx2 binds to specifically sulfated CS of PNNs enwrapping cortical parvalbumin interneurons. Otx2 is then internalized by the interneurons, where it promotes their maturation and consequently the closure of the critical period. In their current paper, the authors discuss how the PNN interplays with Otx2 to regulate visual cortex plasticity and how interfering with this interaction can reopen windows of plasticity in the adult.

The idea that the concentration of specific plasticity-regulatory factors around neurons is controlled by PNNs may be also true for the repulsive axon guidance molecule Semaphorin 3A (Sema3A), as discussed in the review “The Chemorepulsive Protein Semaphorin 3A and Perineuronal Net-Mediated Plasticity” by F. de Winter et al. In this paper, recent data on Sema3A distribution in PNNs in the adult CNS, interaction of this molecule with specific PNN-CS sugars, and changes in Sema3A expression during brain plasticity are reported. It strongly suggests that Sema3A is an important PNN component for regulation of neuronal plasticity.

Emerging evidence implicates ECM/PNNs in the pathophysiology of several neurodevelopmental, neurological, and psychiatric disorders. In the paper “In Sickness and in Health: Perineuronal Nets and Synaptic Plasticity in Psychiatric Disorders,” H. Pantazopoulos and S. Berretta review recent data about PNN abnormalities in psychiatric conditions, with particular focus on schizophrenia, and discuss the hypothesis that ECM/PNN alterations may significantly contribute to synaptic dysfunction, which is a critical pathological component of several brain disorders.

One of the most recent discoveries concerning PNNs is their role in drug addiction and drug-related memories. In the review “Caught in the Net: Perineuronal Nets and Addiction,” M. Slaker et al. address this topic by discussing drug-induced changes in PNNs in brain circuitries underlying drug-related motivation, reward, and reinforcement.

We hope that this special issue will stimulate further studies on gaining a deeper understanding of the role of PNNs in brain physiology and pathology. We believe that a better knowledge of the structure and function of PNNs in physiological and pathological conditions and of the consequences of manipulating the PNN has a strong potential for the development of therapies to enhance neuronal plasticity and functional recovery in a number of CNS conditions, from neurodevelopmental disorders to injury and drug addiction.

*Daniela Carulli* *Daniela Carulli* *Jessica C. F. Kwok* *Jessica C. F. Kwok* *Tommaso Pizzorusso* *Tommaso Pizzorusso*

